# Screening for functional gastrointestinal disorders in preterm infants up to 12 months of corrected age: a prospective cohort study

**DOI:** 10.1007/s00431-024-05451-4

**Published:** 2024-02-12

**Authors:** Yusuf Aydemir, Ozge Aydemir, Meltem Dinleyici, Adviye Cakil Saglik, Demet Cam, Tugba Barsan Kaya, Fuat Emre Canpolat

**Affiliations:** 1 Faculty of Medicine Department of Pediatrics, Division of Gastroenterology and Hepatology, Eskisehir Osmangazi University, Meselik 26040 Eskisehir, Turkey; 2 Faculty of Medicine Department of Pediatrics, Division of Neonatology, Eskisehir Osmangazi University, Eskisehir, Turkey; 3 Faculty of Medicine Department of Pediatrics, Division of Social Pediatrics, Eskisehir Osmangazi University, Eskisehir, Turkey; 4 Neonatal Intensive Care Unit, Dr. Zekai Tahir Burak Womens Health Research and Education Hospital, Ankara, Turkey; 5 Department of Pediatrics, Division of Neonatology, University of Health Science Ankara Bilkent City Hospital, Ankara, Turkey

**Keywords:** Functional gastrointestinal disorders, Prematurity, Rome 4 criteria, Screening

## Abstract

Functional gastrointestinal disorders (FGIDs) are characterized by a variety of symptoms that are frequently age-dependent, chronic, or recurrent and are not explained by structural or biochemical abnormalities. There are studies in the literature reporting different results regarding the relationship between prematurity and FGIDs. The main objective of this study was to compare the frequency of FGIDs between preterm and term infants. The secondary objective was to evaluate whether there was any association between neonatal characteristics and development of FGIDs. A multicenter prospective cohort study that included preterm infants born before 37 weeks of gestation and healthy term infants was carried out. At 1, 2, 4, 6, 9, and 12 months of age, infants were assessed for the presence of FGIDs using the Rome IV criteria. In preterm infants, an additional follow-up visit was made at 12 months corrected age. 134 preterm and 104 term infants were enrolled in the study. Infantile colic, rumination syndrome, functional constipation, and infant dyschezia were more common in preterm infants. Incidence of other FGIDs (infant regurgitation, functional diarrhea and cyclic vomiting syndrome) were similar among preterm and term infants. Preterm infants who are exclusively breastfeed in the first 6 months of life have a lower incidence of infantile colic (18.8% vs 52.1%, p = 0.025). In terms of chronological age, FGIDs symptoms started later in preterm infants; this difference was statistically significant for infantile colic and regurgitation (median age 2 months vs 1 month, p < 0.001).

*   Conclusions*: Preterm infants have a higher prevalence of FGIDs compared with term controls. Therefore, especially if they have gastrointestinal complaints, they should be screened for FGIDs. Possibly due to maturational differences, the time of occurrence of FGIDs may differ in preterm infants. Infantile colic incidence decreases with exclusive breastfeeding.

**Table:** 

**What is Known:** • *The functional gastrointestinal disorders are a very common in infancy.* • *Data on preterm infants with FGIDs are currently very limited.*
**What is New:** • *Preterm infants have a higher incidence of infantile colic, rumination syndrome, functional constipation and infant dyschezia when compared to term infants.* • *Preterm infants who are exclusively breastfed during the first 6 months of life experience a lower incidence of infantile colic.*

**What is Known:**

• *The functional gastrointestinal disorders are a very common in infancy.*

• *Data on preterm infants with FGIDs are currently very limited.*

**What is New:**

• *Preterm infants have a higher incidence of infantile colic, rumination syndrome, functional constipation and infant dyschezia when compared to term infants.*

• *Preterm infants who are exclusively breastfed during the first 6 months of life experience a lower incidence of infantile colic.*

## Introduction

Functional gastrointestinal disorders (FGIDs) are characterized by a variety of symptoms that are frequently age-dependent, chronic, or recurrent and are not explained by structural or biochemical abnormalities [1]. Researchers have been studying the underlying mechanisms of FGIDs for decades, with a focus on changes in gastrointestinal motility and visceral sensory function [2, 3]. Recent researches have focused on a variety of mechanisms, such as the gut-brain axis, dietary factors, genetic issues, infections, problems with the intestinal microbiota, low-grade mucosal inflammation, immune activation, altered intestinal permeability, and issues with bile salt and 5-hydroxytryptamine metabolism [4]. A review of the literature evaluated the prevalence of FGIDs in infants shows wide variability due to the heterogeneity of the diagnostic criteria used in different studies [5]. For several FGIDs, such infantile colic and regurgitation, prematurity is considered to be a risk factor [6, 7]. Few studies have been conducted on the frequency of FGIDs in preterm infants, and conflicting findings have been reported [8–10].

The main objective of this study was to compare the frequency of FGIDs between preterm and term infants. The secondary objective was to evaluate whether there was any association between gestational age, anthropometrics at birth, type of delivery, type of feeding, probiotic use and development of FGIDs.

## Material and methods

This study was performed in line with the principles of the Declaration of Helsinki. Approval was granted by the Ethics Committee of Eskisehir Osmangazi University (Date:11.09.2018/No:05). Informed consent was obtained from the parents of each infant before recruitment. Preterm infants (gestational age less than 37 weeks) and term infants (gestational age equal to or greater than 37 weeks) were included in the study. Preterm infants were recruited between 01.01.2018 and 31.12.2020 from two centers: Neonatal Intensive Care Unit of Eskisehir Osmangazi University Hospital in Eskisehir and Dr. Zekai Tahir Burak Women's Health Research and Education Hospital in Ankara. Preterm infants were enrolled in the study before being discharged from the neonatal intensive care unit. They were then regularly followed through visits at the preterm infant outpatient clinic after discharge up to 12 months corrected age. Inborn term infants were recruited from the same centers shortly after birth and were also followed in regular intervals in the well-baby outpatient clinic up to 12 months of age.

Infants with a history of necrotizing enterocolitis, abdominal surgery, congenital anomalies, alternative feeding routes after discharge, organic gastrointestinal pathologies, and those using medication affecting gastrointestinal motility were excluded from the study.

We conducted in-person interviews with parents and caregivers, evaluating infants to detect the presence of FGIDs using Rome IV criteria at 1, 2, 4, 6, 9, and 12 months of age. Additional follow-up visits were conducted for preterm infants at 12 months corrected age. These FGIDs include infant regurgitation, infant rumination syndrome, cyclic vomiting syndrome, infant colic, functional diarrhea, infant dyschezia, and functional constipation. Evaluations of preterm infants before discharge involved interviews with parents, nurses, and the responsible physician. Follow up of term infants and the preterm infant after discharge were carried out in outpatient clinics through interviews with the parents and responsible physicians. Patients with positive screening results were referred to the pediatric gastroenterology clinic for further evaluation of alarming symptoms and signs for organic GI disorders through history and detailed physical examination. Infants diagnosed with any organic GI disorder including cow’s milk allergy were excluded from the study. We collected the data related to clinical and feeding characteristics of the infants from medical records and through interviews with the parents during follow-up visits. Feeding characteristics include: the day when enteral feeding was started and full enteral feeding was achieved in the NICU in preterm infants, presence of exclusive breast feeding in the first 6 months of life and total duration of breast feeding. A part from feeding characteristics we also evaluated the association between FGIDS and gestational age, being SGA, type of delivery, gender, antibiotic use in neonatal period and probiotic use during infancy.

## Results

**Fig. 1:**
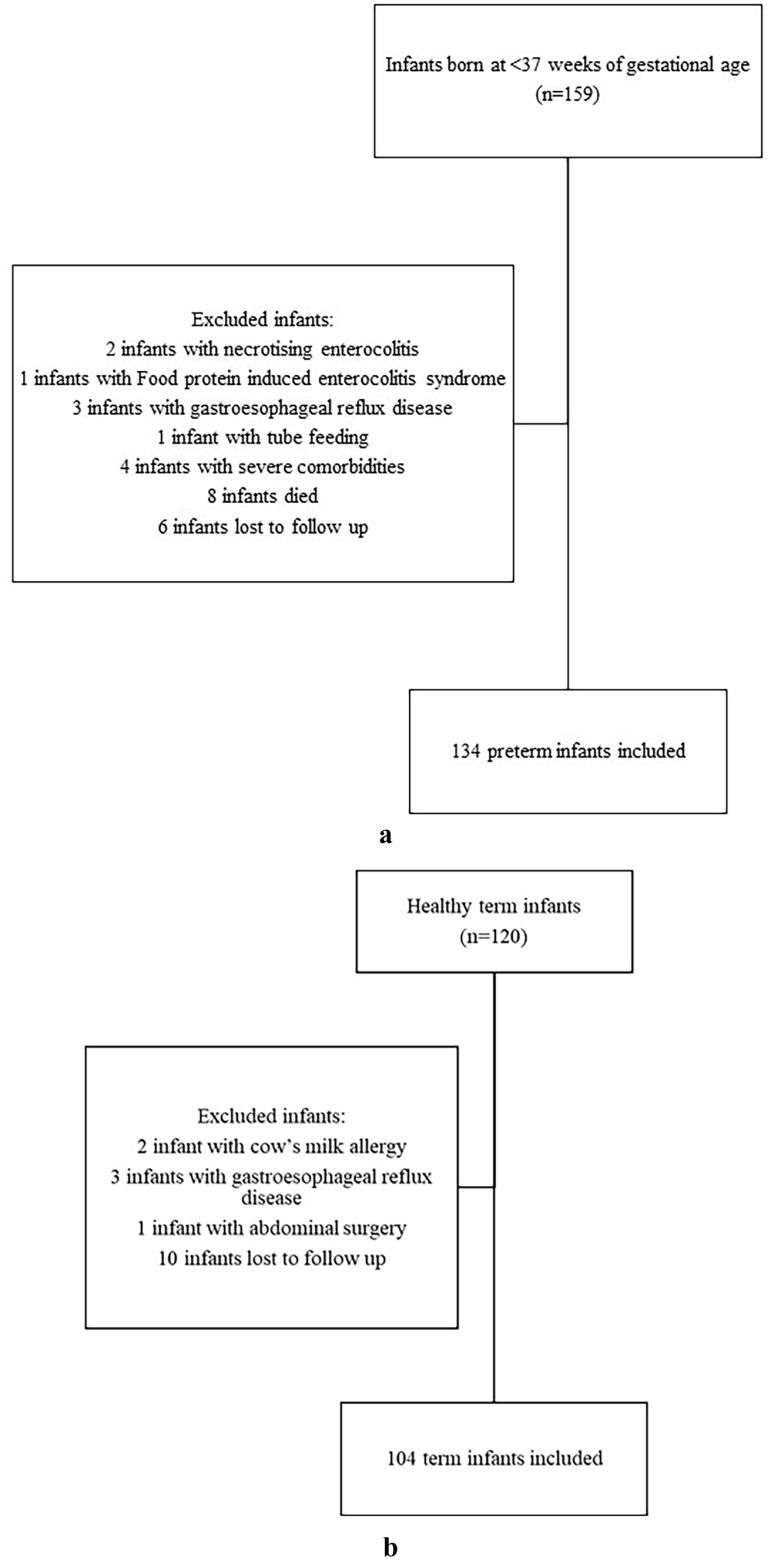
**a** Recruitment flow chart of preterm infants. **b** Recruitment flow chart of term infants

A total of 279 infants were included. Twenty-five preterm and 16 term infants were excluded due to various reasons (Fig. 1a-b). Thus, 238 infants were studied, comprising 134 preterm [60 girls, 74 boys; median gestational age 30 (28–32) weeks] and 104 term infants [53 girls, 51 boys; median gestational age 38 (37–39) weeks]. The preterm group exhibited higher proportions of cesarean delivery (76.1% vs 52.9%, p = 0.01), small-for-gestational-age (SGA) infants (20.2% vs 4.8%, p = 0.001), and use of antibiotics (67.2% vs 2.9%, p < 0.001) and probiotics (37.3% vs 13.5%, p < 0.001). Additionally, the rate of exclusive breastfeeding in the first six months was higher in term infants (46.2% vs 12.7%, p < 0.001) (Table 1).

**Table 1:** Demographic and feeding characteristics, and neonatal histories of the infants involved in the study

**Demographic characteristics**	**Preterm** **(n = 134)**	**Term** **(n = 104)**	***p***
**Gestational age, weeks, median (IQR)**	30 (28–32)	38 (37–39)	< 0.001
**Birth weight, g, median (IQR)**	1310 (950–1726)	3225 (2925–3560)	< 0.001
**Gender, male/female**	60/74	50/51	0.343
**Small for gestational age, n (%)**	24 (20.2)	5 (4.8)	0.001
**Cesarean section, n (%)**	102 (76.1)	56 (52.9)	0.01
**Enteral feedings started, DOL, median (IQR)**	2 (1–3)	1	< 0.001
**Full enteral feedings achieved, DOL, median (IQR)**	18 (9–28)	1	< 0.001
**Exclusive breastfeeding in the first 6 months, n (%)**	17 (12.7)	48 (46.2)	< 0.001
**Breastfeeding at 12 months of age, n (%)**	42 (31.3)	59 (56.7)	< 0.001
**Duration of breast feeding, months mean ± SD**	7,2 ± 4,2	9,4 ± 3,3	< 0.001
**Antibiotic use in the neonatal period, n (%)**	90 (67.2)	3 (2.9)	< 0.001
**Probiotic use, n (%)**	50 (37.3)	14 (13.5)	< 0.001

*IQR* Inter quartile range, *DOL* Day of life

At least one FGID was found in 110 (82.1%) preterm infants and 57 (54.8%) term infants (p < 0.001). Preterm infants exhibited higher rates of infantile colic (47.8% vs 20%, p < 0.001), rumination syndrome (15.7% vs 1.9%, p = 0.001), functional constipation (32% vs 5.8%, p < 0.001), and infant dyschezia (20.1% vs 1.9%, p < 0.001) compared to term infants. Incidences of other FGIDs (infant regurgitation, functional diarrhea, and cyclic vomiting syndrome) were similar between the groups (Table 2).

**Table 2:** Incidences of FGIDs among preterm and term infants

**Functional gastrointestinal disorders**	**Preterm** **(n = 134)**	**Term** **(n = 104)**	***p***
**At least 1 FGIDs (%)**	110 (82.1)	57 (54.8)	< 0.001
**Infant regurgitation (%)**	77 (57.5)	49 (47.1)	0.113
**Rumination syndrome (%)**	21 (15.7)	2 (1.9)	0.001
**Cyclic vomiting syndrome (%)**	2 (1.5)	0	0.506
**Infantile colic (%)**	64 (47.8)	21 (20)	< 0.001
**Functional diarrhea (%)**	3 (2.2)	0	0.259
**Infant dyschezia (%)**	28 (20.1)	2 (1.9)	< 0.001
**Functional constipation (%)**	43 (32.1)	6 (5.8)	< 0.001

*FGIDs* Functional Gastrointestinal disorders

The incidence of functional constipation increased as gestational age decreased in the preterm infants (p = 0.026). However, the occurrences of other FGIDs did not show significant variations across different gestational age groups in preterm infants (Table 3).

**Table 3:** Incidences of FGIDs among preterm infants according to gestational age at birth

**FGIDs**	** < 28 weeks**	**28–30 weeks**	**31–33 weeks**	**34–36 weeks**	***p***
**At least 1 FGIDs (%)**	29 (90.6)	36 (78.3)	28 (77.8)	17 (85)	0.461
**Infant regurgitation (%)**	23 (71.9)	24 (52.2)	21 (58.3)	9 (45)	0.217
**Rumination syndrome (%)**	6 (18.8)	8 (17.4)	6 (16.7)	1 (5)	0.575
**Cyclic vomiting syndrome (%)**	0	1 (2.2)	1 (2.8)	0	1
**Infantile colic (%)**	16 (50)	19 (41.3)	16 (44.4)	14 (70)	0.185
**Functional diarrhea (%)**	0	3 (6.5)	0	0	0.128
**Infant dyschezia (%)**	6 (18.8)	9 (19.6)	8 (22.2)	5 (25.5)	0.929
**Functional constipation (%)**	15 (46.8)	17 (37)	9 (25)	2 (10)	0.026

*FGIDs* Functional Gastrointestinal disorders

Gestational age, gender, SGA, cesarean section, exclusive breastfeeding in the first six months, initiation of enteral feedings later than 2 days of life (DOL-2), and achieving full enteral feedings later than DOL-14 were investigated as potential risk factors for FGIDs in preterm infants. The absence of exclusive breastfeeding in the first six months was identified as a significant risk factor for FGIDs in preterm infants [OR 9.14, (1.956–42.67, 95% CI), p = 0.005] (Table 4). Individual FGIDs were also evaluated separately for risk factors. Being SGA [OR 0.33, (0.11–0.98, 95% CI), p = 0.046] and using probiotics [OR 0.29, (0.12–0.72, 95% CI), p = 0.007] were associated with a lower risk of infant regurgitation. Exclusively breastfed preterm infants had a lower incidence of infantile colic (18.8% vs. 52.1%, p = 0.025). The absence of exclusive breastfeeding in the first 6 months of life was associated with nearly a 20-fold increase in infantile colic [ (2.29–180.7, 95% CI), p = 0.007]. Additionally, gestational age < 28 weeks was identified as an independent risk factor for functional constipation [OR 2.86, (1.02–8.06, 95% CI), p = 0.047]. The investigated potential risk factors did not show a significant association with the incidence of other FGIDs when evaluated separately.

**Table 4:** Multivariate analysis of risk factors for FGIDs in preterms

**Risk factors**	**Multivariate**		
	**OR**	**95% CI**	***p***
**Gestational age < 28 weeks**	3.45	0.614–19.41	0.16
**Male gender**	0.89	0.295–2.71	0.843
**SGA**	0.34	0.88–1.31	0.117
**Cesarean section**	2.51	0.912–15.3	0.092
**Enteral feeding started later than DOL 2**	0.54	0.158–1.85	0.327
**Full enteral feeding achieved later than DOL 14**	0.59	0.154–2.24	0.437
**Breast milk dominant feeding in the NICU**	0.39	0.119–1.32	0.133
**Absence of exclusive breast feeding in the first 6 months**	9.14	1.956–42.67	0.005
**Probiotics**	0.62	0.206–1.86	0.392

*SGA* Small for gestational age, *DOL* Day of life, *NICU* Neonatal intensive care unit

**Fig. 2:**
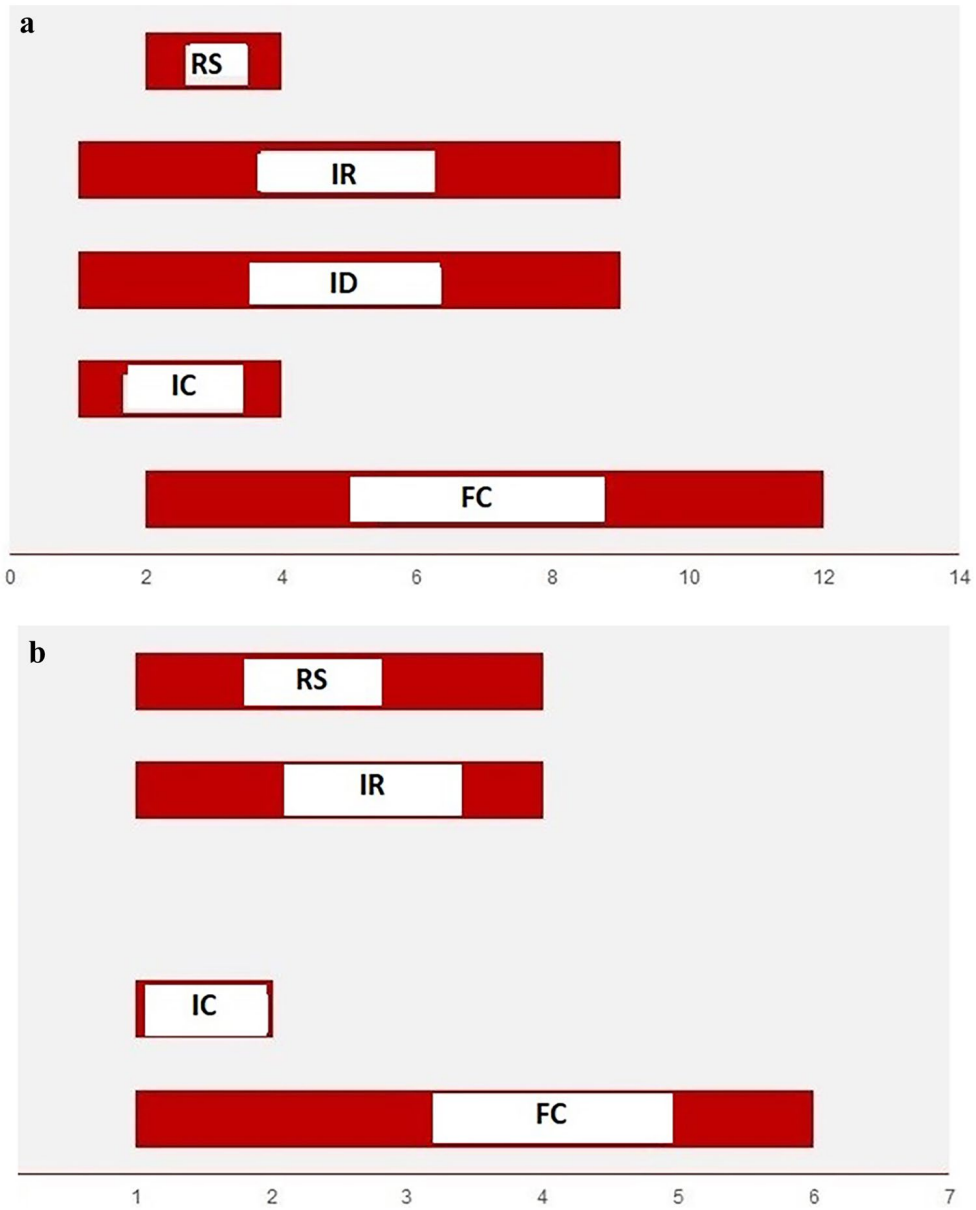
**a** Time of onset of FGIDs in preterm infants by chronological age (months). RS: Rumination syndrome, IR: Infant regurgitation, ID: Infant dyschezia, IC: Infantile colic, FC: Functional constipation. **b** Time of onset of FGIDs in term infants by chronological age (months). RS: Rumination syndrome, IR: Infant regurgitation, ID: Infant dyschezia, IC: Infantile colic, FC: Functional constipation

FGIDs symptoms started at an older chronological age in preterm infants compared to term infants; this difference was statistically significant for infantile colic and regurgitation. (median 2 months vs 1 months of age, p < 0.001) (Fig. 2a-b).

## Discussion

Functional gastrointestinal disorders seem to manifest in a significant proportion of infants younger than 12 months. In a meta-analysis study, the reported prevalence exhibited wide variation, ranging from 2 to 73% (30 studies) for infantile colic, 3% to 87% (12 studies) for infant regurgitation, and 0.1% to 39% (8 studies) for functional constipation. The prevalence of functional diarrhea and dyschezia consistently remained below 10% [5]. The wide variability can be attributed to differences in the diagnostic criteria used and study designs. Few studies have specifically focused on the frequency of FGIDs in preterm infants, and among these, even fewer have a longitudinal design, making direct comparisons challenging.

In a cross-sectional Brazilian study evaluating the frequency of FGIDs in the first 2 years of life, no significant difference was found between preterm and term infants [11]. However, a separate cross-sectional study from Türkiye reported a higher prevalence of regurgitation, infantile colic, and dyschezia in preterm infants during the first 12 months compared to term infants [12]. The frequencies of both overall and individual FGIDs were considerably lower than observed in our study. Cross-sectional studies may underestimate the true frequency of FGIDs because the onset and peak time of symptoms vary for each FGID. For example, infantile colic symptoms typically commence in the 4th to 6th week and gradually diminish by the time the baby is 3 to 4 months old. If a baby is included in the study after 6 months, the critical period for infantile colic has already passed. Longitudinally designed studies have the potential to provide a more accurate prediction of FGIDs incidence during infancy. In line with this, a prospective cohort study from Italy reported a higher incidence of FGIDs, with preterm infants being more commonly affected (86%) compared to term infants (73%) [8]. Similarly, in our study, we observed a higher overall incidence of FGIDs in preterm infants compared to full-term controls, with at least one FGID identified in 82.1% of preterm infants. Preterm infants are frequently exposed to various prenatal and postnatal factors that impact growth, development, intestinal microbiome, and various noxious experiences in the intensive care nursery, potentially affecting sleep patterns and the processing of pain and other stimuli. The significantly higher rate of FGIDs in preterm infants might also be influenced by parental anxiety related to the stress associated with preterm deliveries, leading to an overreporting of symptoms. In addition, the high incidences we observed might be attributed to specific characteristics within our cohort, such as a higher rate of cesarean deliveries and lower rates of breastfeeding. Moreover, our utilization of face-to-face assessments and a longitudinal study design may have significantly enhanced the diagnostic precision for FGIDs in preterm infants.

In our study, regurgitation (57.5%) emerged as the most common FGID in preterm infants. The Italian study, akin to ours, identified regurgitation (45.7%) as the second most common FGID [8]. In both investigations, regurgitation was more frequent in preterm infants compared to term infants. Notably, the regurgitation rate reported in the Brazilian study was lower in term newborns (35.1% vs 15.6%) [11]. Despite their belief that regurgitation was associated with type of feeding, as the term infants had higher rates of breastfeeding, multivariate analysis failed to support this connection. Furthermore, our study revealed that infants using probiotics exhibited a lower frequency of regurgitation. Previous research suggests that specific probiotic strains, particularly *L. reuteri* DSM 17938, may alleviate functional symptoms in the stomach and esophagus by promoting gastric emptying and reducing the frequency of regurgitation events per day [13]. Vandenplas et al. demonstrated that a symbiotic infant formula supplemented with B. lactis and fructo-oligosaccharides resulted in a decreased incidence of daily regurgitations [14]. In our study, being SGA was linked to a lower risk of IR. The association of SGA with FGIDs is largely unknown. Milidou et al. observed an increased risk of infantile colic in preterm and SGA infants in a large cohort study conducted in Denmark [6]. Unfortunately, their investigation did not address other FGIDs. An inadequate intrauterine environment, leading to growth restriction and preterm birth, may influence the function of various organ systems later in life, as indicated by the theory of fetal organ programming. Decreased intestinal perfusion secondary to the redistribution of blood to vital organs in response to chronic hypoxia in intrauterine growth-restricted fetuses often leads to feeding intolerance and, sometimes, necrotizing enterocolitis after birth. Intestinal hypoperfusion experienced in utero might also be related to more complex functional problems later in life. This could potentially explain the associations between preterm birth, being SGA, and the occurrence of FGIDs.

Infantile colic, accounting for 47.8%, emerged as the second most common FGID in our cohort, with a notably higher frequency among preterm infants. This trend aligns with findings from the Italian study, which also identified a correlation between prematurity and neonatal antibiotic use [8]. Dysbiosis and lactase deficiency have been proposed as potential factors contributing to the development of infantile colic [15, 16]. In our investigation, we demonstrated that exclusive breastfeeding for the first 6 months significantly reduced the risk of infantile colic. This protective effect could be attributed to breast milk's role in fostering the development of a healthy microbiota [17]. Moreover, infants who are not exclusively breastfed during this initial period not only experience fewer benefits from the advantageous and protective effects of breast milk but also encounter new antigens through complementary foods at an earlier stage. In line with our findings, a previous study reported a higher incidence of early supplementary feeding among infants with FGIDs aged ≤ 6 months [12]. Premature exposure to various antigenic stimuli could be an additional factor contributing to the development of infantile colic.

We observed a higher frequency of constipation in preterm infants compared to term infants (32.1% vs. 5.8%). In addition, our findings indicated that constipation was more prevalent as gestational age decreased. The incidence of constipation did not differ between term and preterm infants in the Brazilian and Italian trials [8, 11]. On the other hand, a significant rate of laxative use (approximately 40%) was noted among preterm infants up to six months of age in a study conducted in Denmark, involving 286 preterm children[18]. Small intestinal motility, as evidenced by intestinal transit time studies, appears to be immature before 34 weeks of gestational age and improves as gestational age approaches term. However, our understanding of colonic motility is limited. Various colonic motility patterns have been described in children and adults through the use of high-resolution colonic manometry. Despite these advancements, the timing of colonic motor pattern development in gestation and its evolution in preterm infants remain unknown to date [19].

A study conducted in Colombia found that the prevalence of functional diarrhea was 1.9% among infants in the first year of life, irrespective of gestational age at birth [20]. There are only a few studies that have assessed functional diarrhea in preterm infants, and their reported prevalences ranged from 3.3% to 4.6%, slightly higher than what we observed [8, 11]. In our study, while functional diarrhea was not identified in term infants, it was observed in 2.2% of preterm infants.

Dyschezia is a functional condition that has received limited research attention, with few studies in the literature. It occurs due to the inability to coordinate the relaxation of the pelvic floor during bowel movements with the increase in abdominal pressure. According to a questionnaire-based study involving 1447 mothers, the prevalence of dyschezia in the first year of life is reported as 2.4% [21]. Our observation of dyschezia frequency in term newborns aligns with the existing literature. There is a hypothesis that preterm babies may experience a delay in this mechanism, leading to an increased prevalence of dyschezia. Consistent with this hypothesis, our research found a significantly higher rate of dyschezia in preterm infants.

Rumination is the habitual regurgitation of stomach contents into the mouth for the purpose of self-stimulation [22]. A recent questionnaire-based study showed a prevalence of 1.9% [21]. There is a scarcity of data regarding the prevalence of rumination syndrome in preterm infants within the existing literature. We observed a rumination syndrome incidence of 1.9% in term infants, consistent with the literature. However, in preterm infants, this rate increased significantly to 15.7%. Rumination historically has been considered a self-stimulatory behaviour that arises in the context of longstanding social deprivation [1]. The higher rate of rumination syndrome observed in preterm infants might be attributed to prolonged hospital stays, lack of stimuli, and reduced social interaction.

Cyclic vomiting syndrome is characterized by stereotypical and repeated episodes of vomiting lasting from hours to days with intervening periods of return to baseline health [23]. Cyclic vomiting syndrome occurs from infancy to middle-age adulthood, with a peak prevalence between 2 and 7 years [24]. The population-based survey study from Colombia found the prevalence of CVS to be 3.8% in infants < 1 year and 6.1% in children from 1–4 years of age [20]. In our study, term infants did not exhibit cyclic vomiting, whereas it was observed in only 1.5% of preterm infants.

We investigated the potential influence of neonatal characteristics, including gestational age, birth weight, delivery method, feeding type, and probiotic use, on the emergence of FGIDs. Exclusive breastfeeding in the initial six months emerged as a protective factor, significantly reducing the risk of functional gastrointestinal disorders, particularly infantile colic. The intricate mechanisms behind this phenomenon involve the establishment and maintenance of a balanced and diverse gut microbiota. Exclusive breastfeeding fosters the growth of beneficial bacterial strains, such as Bifidobacterium and Lactobacillus, which play pivotal roles in immune modulation and the production of short-chain fatty acids [25, 26]. These microbial activities contribute to a well-regulated gastrointestinal environment. Moreover, breast milk serves as a rich source of bioactive compounds, including immunoglobulins, cytokines, and oligosaccharides, all of which contribute to the development and maturation of the infant's immune system and the overall health of the gut [27]. The anti-inflammatory properties of breast milk may further mitigate the occurrence of gastrointestinal disturbances, including infantile colic [28, 29].

The onset and duration of each FGID were defined for term infants, but the natural course of these disorders remains poorly described for preterm infants. Neural maturation of gastrointestinal motility, including functions like swallowing, sphincter functions, peristalsis, intestinal, and colonic motility, is dependent on gestational/ postmenstrual age in preterms [19] Thus, we propose that, due to maturational variances, the onset timing of FGIDs may differ in preterm infants. While screening term-born infants up to 12 months of age, we continue follow-up until 12 months corrected age in preterm-born infants in our cohort. For the first time, we have demonstrated that symptoms of FGIDs commence at an older chronological age in preterm-born infants. This difference is statistically significant, particularly for infantile colic and regurgitation.

While our study presents valuable insights, it is essential to acknowledge certain limitations. One notable constraint is the relatively small number of infants included in each FGIDs category, conforming to the age ranges specified by the Rome IV criteria. On a positive note, our study boasts several strengths, including its multicenter and longitudinal design, the comprehensive analysis of various neonatal factors, the utilization of the latest version of the Rome criteria (Rome IV) for FGIDs classification, and the inclusion of both preterm and term neonates. Notably, these infants were enrolled at birth and underwent prospective follow-up until 12 months of age through face-to-face consultations and physical examinations.

## Conclusion

In conclusion, we found that preterm infants have a higher prevalence of FGIDs when compared with age- and sex-matched term infants. Infantile colic, rumination syndrome, functional constipation and infant dyschezia were more common in preterm infants. Exclusively breastfed preterm infants during the first 6 months of life experience a lower incidence of functional gastrointestinal disorders, particularly infantile colic. FGIDs symptoms started later in preterm infants; this difference was statistically significant for infantile colic and regurgitation. Preterm infants, especially complaining of gastrointestinal symptoms, should be screen for FGIDs.

## Data Availability

Our data is available upon request, and we are committed to providing access to interested researchers for the purpose of scientific inquiry.

## Abbreviations

CVS: Cyclic vomiting syndrome
DOL: Day of life
FGIDs: Functional gastrointestinal disorders
FC: Functional constipation
FD: Functional diarrhea
IC: Infantile colic
ID: Infant dyschezia
IR: Infant regurgitation
IRS: Infant rumination syndrome
SGA: Small for gestational age
